# Disparities in Preventive Dental Care Among Children in Georgia

**DOI:** 10.5888/pcd14.170176

**Published:** 2017-10-26

**Authors:** Shanshan Cao, Monica Gentili, Paul M. Griffin, Susan O. Griffin, Nicoleta Serban

**Affiliations:** 1 Industrial and Systems Engineering School, Georgia Institute of Technology, Atlanta, Georgia; 2 Industrial Engineering, University of Louisville, Louisville, Kentucky; 3 Regenstrief Center for Healthcare Engineering, Purdue University, West Lafayette, Indiana; 4 Division of Oral Health, Centers for Disease Control and Prevention, Atlanta, Georgia

## Abstract

**Introduction:**

We compared access to preventive dental care among low-income children eligible for public dental insurance to access among children with private dental insurance and/or high family income (>400% of the federal poverty level) in Georgia, and the effect of policies toward increasing access to dental care for low-income children.

**Methods:**

We used multiple sources of data (eg, US Census, Georgia Board of Dentistry) to estimate, by census tract, measures of preventive dental care access in 2015 for children aged 0 to 18 years. Measures were percentage of met need, 1-way travel distance to a dentist, and scarcity of dentists. We used an optimization model to estimate access, quantify disparities, and evaluate policies.

**Results:**

About 1.5 million children were eligible for public insurance; 600,000 had private insurance and/or high family income. Across census tracts, average met need was 59% for low-income children and 96% for high-income children; for rural census tracts, these values were 33% and 84%, respectively. The average 1-way travel distance for all census tracts was 3.7 miles for high-income and/or privately insured children and 17.2 miles for low-income children; for rural census tracts, these values were 11.6 and 32.9 miles, respectively. Increasing dentists’ acceptance of public insurance–eligible children increased met need more in rural areas than in urban areas. To achieve 100% met need in rural tracts, however, an 80% participation rate among dentists would be required.

**Conclusion:**

Across census tracts, high-income children had better access to preventive dental care than low-income children had. Identifying tracts with disparities in access could result in more efficient allocation of public health dental resources.

## Introduction

Children living in poverty are more than twice as likely to have untreated tooth decay as children with family incomes greater than 200% of the federal poverty level (FPL) (25% vs 12%) (1). Tooth decay, if left untreated, can lead to problems in eating, speaking, and learning (2). Strong evidence demonstrates the effectiveness of preventive dental care services (3), and increasing low-income children’s access to these services is a national health goal (4). A major barrier to poor children not receiving dental care is difficulty in finding a dentist who accepts Medicaid (5). Policies and programs aimed at increasing access to preventive dental care (eg, increasing the number of dental care providers or providing services in schools) are typically implemented locally.

The objective of this study was to estimate 3 measures of local access to preventive dental care services – percentage of met need for preventive dental services, 1-way travel distance to a dentist, and dentist scarcity – by census tract among children in Georgia. We compared local access for 2 groups: children eligible for public dental insurance and children with private dental insurance and/or high family income. We also estimated these measures separately for rural and urban tracts. Finally, we examined the effect of increasing dentists’ participation in public insurance programs (Medicaid and Children’s Health Insurance Program [CHIP]) on preventive dental care access in both groups.

## Methods

We used data from the US Census and the American Community Survey (6) to compare access to preventive dental services for Georgia children aged 0 to 18 years in 2 groups: children living in households with family incomes less than or equal to 247% of the FPL (the income threshold for Medicaid/CHIP eligibility [7]), hereinafter referred to as publicly insured children, and 2) children in families with an income greater than 400% of the FPL, hereinafter referred to as privately insured children. We assumed the latter group would have private insurance or be able to afford out-of-pocket expenses.

We calculated 3 measures of access for each census tract. We calculated percentage of met need as the total met need divided by pediatric need for preventive dental care services. Met need refers to the need served within state access standards (8), which specify the maximum distance to be traveled in rural or urban areas to reach a provider for people using a private vehicle or using public transportation. The state access standards are 30 miles or 30 minutes for urban communities and 45 miles or 45 minutes for rural communities. Higher values indicate smaller proportions of children who need to travel longer distances than the distances specified by state access standards to reach an available provider. We calculated travel distance as the average distance in miles a child must travel from his or her residence 1 way to visit the dentist. Higher values indicate larger travel distances. We computed travel distance by using street networks indicated by Esri’s ArcMap GIS (geographic information system) version 10.3.1 software. We calculated provider scarcity as the patient caseload served by dentists divided by maximum patient caseload capacity. Higher values indicate greater scarcity of dentists.

We also designated census tracts as served, underserved, or unserved according to the proportion of children with unmet need within the state access standards or the proportion of uninsured children in households that cannot afford dental care: 10% or less (served), 10% to 50% (underserved), and more than 50% (unserved).

We estimated these measures across all census tracts in Georgia and separately across rural census tracts only (located in counties with population <35,000) and urban tracts only (population ≥35,000) (9). To estimate need for pediatric preventive dental services, we used a published methodology (10) to estimate the number of dental care provider hours required to provide preventive dental services at a frequency recommended by the American Academy of Pediatric Dentistry (11) and the American Dental Association (12). Recommended services and frequency of delivery depend on a child’s age and risk for caries.

We obtained a list of Georgia dentists and their practice addresses from the 2015 Georgia Board of Dentistry. We used their taxonomy code (2015 National Plan and Provider Enumeration System) to identify providers of preventive dental services to children. We geocoded the addresses of individual dentists and computed street-network distances between dentists’ addresses and census tract centroids by using Texas A&M Geocoding Services (13). Maximum capacity for preventive dental care for children per dentist was estimated according to existing estimation procedures (10). The proportion of provider capacity allocated to prevention was based on the distribution of services as defined in the Medical Expenditure Panel Survey in units of time (14).

To estimate the number of dentists accepting public insurance in each census tract, we used data from InsureKidsNow.gov (IKN). By using an approach similar to that used by the American Dental Association (15), we matched dentists recorded as accepting public insurance in the IKN database with all dentists in the Georgia Board of Dentistry list by using fuzzy logic, after removing repeats in the IKN data and accounting for both individual dentists and dental care offices. For dental care offices, we assumed all dentists who were identified in an office that was recorded in the IKN database accepted public insurance.

We used 2012 Medicaid Analytic Extract claims data obtained from the Centers for Medicare & Medicaid Services to estimate the distribution of caseload capacity allocated by each dentist for publicly insured children; these data accounted for excess capacity attributable to no-shows and potential underutilization. This study was approved by Centers for Medicare & Medicaid Services data use agreement no. 23621 and by the institutional review board of the Georgia Institute of Technology (protocol no. H11287). 

To estimate access, we used an optimization model (16) that matched dental care supply and dental care need under the following set of constraints:

- Supply: the number of patients assigned to each dentist does not exceed the maximum caseload capacity for pediatric preventive care (ie, provider scarcity ≤1);
- Public insurance acceptance: the number of assigned publicly insured patients does not exceed the provider’s public insurance caseload;
- Patient’s travel mobility: the patient’s travel distance does not exceed Georgia guidelines on access standards (17). The maximum distance for patients with personal vehicles is 30 miles in urban areas and 45 miles in rural areas (17). For patients without a private vehicle who must use an alternative means of transportation, we set a maximum distance threshold of 15 miles (45 minutes of travel time) for rural census tracts and 8 miles (30 minutes of travel time) for urban tracts.

The optimization model was based on the assumption that patients prefer providers who are nearer rather than farther to their residence, so the model’s objective was to minimize the total distance traveled to reach dentists by publicly insured and privately insured children. We did not include uninsured children from families with incomes between 247% and 400% of the FPL directly in the optimization model because they were assumed not to have access to public insurance or to have the money necessary to pay for dental care out of pocket. 

The model determined the number of children in the 2 study populations for each census tract assigned to a dentist’s location according to the aforementioned constraints. Need within a census tract could be assigned to different dentists; unmet need within a census tract occurs when the total provider capacity in the census tract is not enough to satisfy all the need. Because many dentists do not accept patients using public insurance, our model assigned privately insured and publicly insured children separately. To account for uncertainty in the estimates of provider caseload and in the proportion of children with a greater need for preventive dental care (ie, high-risk children), we ran 65 microsimulations that simultaneously sampled from these parameters. 

We defined a disparity as the absolute difference in access between publicly insured children (low family income) and privately insured children (insured and/or high family income). Using a simultaneous inference approach (18), we identified census tracts with poorer access than various disparity thresholds at the significance level of .05. For travel distance, we tested the disparity thresholds of 2, 6, 8, and 10 miles. For provider scarcity, we tested the disparity thresholds of 0, 0.1, 0.2, and 0.3. We selected these thresholds because we believed they were reasonable.

We examined the effect of various acceptance rates (ranging from 20% to 80%) of public insurance for children among dentists on our 3 access measures. To do this, we first set the acceptance rate to a given value and then sampled the public insurance caseload separately for dentists in urban tracts (caseload ranged from 35% to 50%) and dentists in rural tracts (caseload ranged from 55% to 65%). Similarly, we varied the caseload capacity of dentists accepting public insurance patients from 20% to 75% and set the maximum allowed travel distance to dentists for families owning a vehicle from 30 miles to 60 miles (for both rural and urban census tracts). Further details on all methods are available at https://healthanalytics.gatech.edu/publications/journal-papers.

## Results

**Figure 1:**
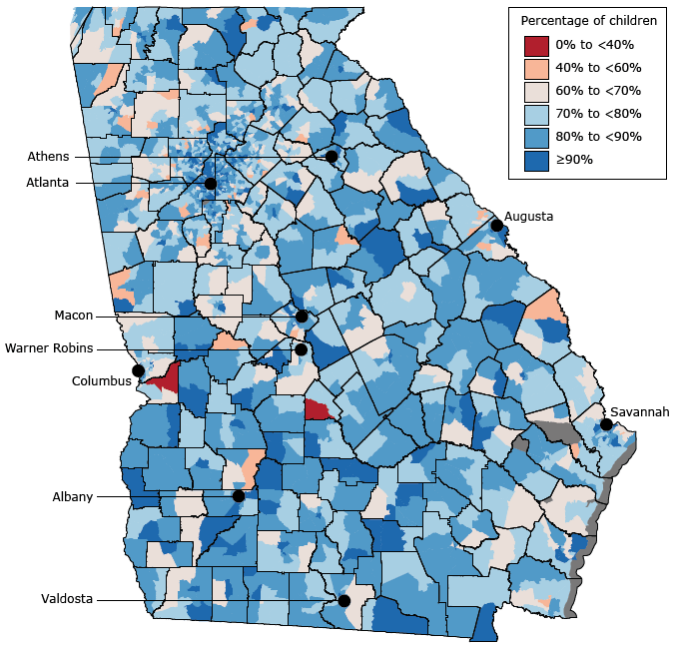
Percentage of children with financial access to preventive dental care in each census tract. Financial access is the percentage of children who either are eligible for public insurance or have the ability to afford dental care through commercial insurance or ability to pay out-of-pocket.

Among the 4,123 dentists who provided preventive dental care to children, 27.9% accepted public insurance. Among Georgia’s approximately 2.6 million children, the estimated number of publicly insured children was 1.5 million, and the number of privately insured children was 600,000. The state has 1,969 census tracts (1,527 urban) in 159 counties (50 urban). The number of publicly insured children was 1,183,470 in urban census tracts and 309,813 in census tracts. The number of privately insured children was 536,043 in urban census tracts and 68,194 in rural census tracts. Both rural and urban census tracts had low percentages of children with financial access to preventive dental care; few census tracts had a percentage greater than 90% (Figure 1). 

The state average (10th–90th percentile) met need for publicly insured children was 0.59 (0–1.00) and for privately insured children was 0.96 (0.90–1.00) (Table 1). In rural areas, these values were 0.33 for publicly insured and 0.84 for privately insured children, and in urban areas, 0.67 for publicly insured and 0.99 for privately insured children. The average travel distance for publicly insured children was 17.2 miles and for privately insured children 3.7 miles. In rural areas, the average travel distance was 32.9 miles and 11.6 miles, and in urban areas 12.6 miles and 1.5 miles for publicly and privately insured children, respectively. The average provider scarcity for publicly and privately insured children was 0.70 and 0.45, respectively. In rural areas, the average provider scarcity was 0.88 and 0.50 and in urban areas 0.65 and 0.43 for publicly and privately insured children, respectively.

**Table 1:** Average Values (10th–90th Percentile) for 3 Measures of Access to Preventive Dental Care Across 65 Microsimulations, by Type of Insurance and Type of Census Tract (Rural or Urban), Georgia, 2015

Measure of Access/Type of Census Tract	Entire State Population^a^	Type of Insurance	
		Public	Private
**Percentage of met need^b^**			
All	0.67 (0.14–1.00)	0.59 (0–1.00)	0.96 (0.90–1.00)
Rural	0.42 (0–0.92)	0.33 (0–0.89)	0.84 (0–1.00)
Urban	0.74 (0.26–1.00)	0.67 (0–1.00)	0.99 (0.99–1.00)
**Travel distance,^c^ mi**			
All	14.4 (0.52–36.43)	17.2 (1.1–45.0)	3.7 (0.02–7.3)
Rural	29.26 (7.95–45.00)	32.9 (10.3–45.0)	11.6 (0.6–45.0)
Urban	10.12 (0.34–23.50)	12.62 (0.74–30.00)	1.46 (0.01–3.56)
**Scarcity of providers^d^**			
All	0.67 (0.38–0.95)	0.70 (0.39–1.00)	0.45 (0.05–0.91)
Rural	0.82 (0.57–1.00)	0.88 (0.65–1.00)	0.50 (0.09–1.00)
Urban	0.63 (0.35–0.91)	0.65 (0.38–1.00)	0.43 (0.04–0.89)

a Entire population in Georgia is represented by 1,969 census tracts (1,527 urban and 442 rural).

b Calculated as the total met need (the need served within state access standards [8]) divided by pediatric need for preventive dental care services. Higher values indicate smaller proportions of children who need to travel longer distances than the distances specified by state access standards to reach an available provider.

c Calculated as the average distance in miles a child must travel from his or her residence 1-way to visit the dentist. Higher values indicate larger travel distances.

d Calculated as the patient caseload served by dentists divided by maximum patient caseload capacity; higher values indicate greater scarcity of dentists.

Assuming a caseload capacity ranging from 35% to 50% in urban census tracts and from 55% to 65% in rural census tracts, we found that 6% of the census tracts were served, 57% were underserved, and 37% were unserved (Table 2).

**Table 2:** Mean Percentage (Range) of Census Tracts^a^, by Level of Preventive Dental Care Service and by Type of Census Tract (Urban or Rural), Across 65 Microsimulations^b^, Georgia, 2015

Type of Census Tract	Level of Preventive Dental Care		
	Served^c^	Underserved^d^	Unserved^e^
All	6 (2–8)	57 (56–60)	37 (36–38)
Urban	8 (3–10)	64 (62–68)	29 (27–31)
Rural	1 (0–2)	35 (32–38)	64 (61–67)

a Entire population of Georgia is represented 1,969 census tracts (1,527 urban and 442 rural).

b In microsimulations, capacity ranged between 35% and 50% for urban communities and between 55% and 65% for rural communities.

c Met need ≥90%.

d Met need from 50% to 90%.

e Met need <50%.

The difference in travel distance between publicly insured children and privately insured children was greater than 2 miles for 72% of the census tracts and greater than 10 miles for 38% of the census tracts (Table 3). The difference in provider scarcity between publicly insured children and privately insured children was greater than 0 in 68% of census tracts and greater than 0.3 in 16% of census tracts.

**Table 3:** Absolute Difference^a^ in Access Measure of Preventive Dental Care Between Publicly Insured Children and Privately Insured Children at Multiple Thresholds of Met Need, Georgia, 2015^b^

Type of Census Tract	Travel Distance, mile				Scarcity of Providers^c^			
	2	6	8	10	>0	>0.1	>0.2	>0.3
All	1,399 (72)	1,104 (56)	934 (48)	749 (38)	1,321 (68)	919 (47)	612 (31)	307 (16)
Urban	1,095 (78)	842 (76)	691 (74)	530 (71)	1,009 (76)	684 (74)	451 (74)	200 (65)
Rural	304 (22)	262 (24)	243 (26)	219 (29)	312 (24)	235 (26)	161 (26)	107 (35)

a For example, the difference in travel distance between publicly insured children and privately insured children was greater than 2 miles for 72% of the census tracts and greater than 10 miles for 38% of the census tracts.

b All values are number (percentage).

c The difference in provider scarcity was greater than 0 in 68% of census tracts and greater than 0.3 in 16% of census tracts. Scarcity was calculated as the patient caseload served by dentists divided by maximum patient caseload capacity; higher values indicate greater scarcity of dentists.

**Figure 2:**
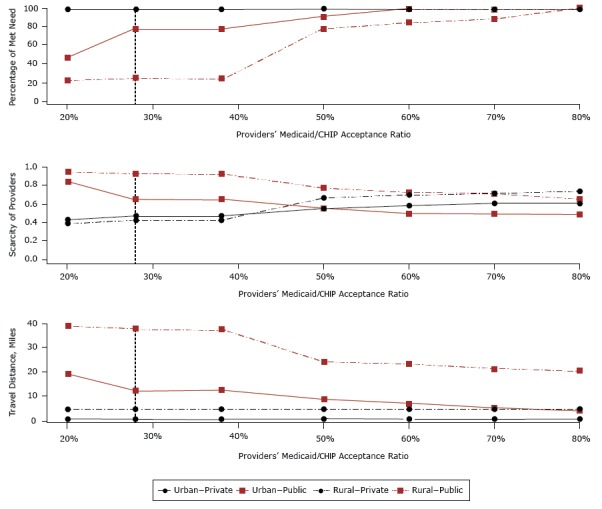
Median values of the percentage of met need, travel distance, and scarcity of dentists in rural and urban census tracts, by dentists’ Medicaid/CHIP acceptance ratio. Scarcity was calculated as the patient caseload served by dentists divided by maximum patient caseload capacity; higher values indicate greater scarcity of dentists. The vertical dashed line at 28% represents the current rate of providers participating in public insurance programs. Abbreviation: CHIP, Children’s Health Insurance Program. **Table:** 

Access to preventive dental care increased among publicly insured children as dentists’ participation in public insurance increased (Figure 2). For a provider participation rate of 20%, the median met need was 30.5%, provider scarcity was 0.86, and the median travel distance was 23.4 miles. To achieve 100% median met need, a provider participation rate of 80% would be required. This increase from 20% to 80% would also result in a decrease in median travel distance to 5.6 miles and a provider scarcity of 0.52. For an increase in provider participation rate from 20% to 80% in rural tracts, the median met need increased from 21.7% to 100%, provider scarcity decreased from 0.94 to 0.65, and the median travel distance decreased from 38.9 miles to 20.2 miles. In urban tracts, the median met need increased from 46.7% to 100%, provider scarcity decreased from 0.83 to 0.47, and the median travel distance decreased from 19.2 miles to 3.8 miles.

Measure of Access by Dentists’ Medicaid/CHIP Acceptance Ratio	Rural–Private	Rural–Public	Urban–Private	Urban–Public
Percentage of met need				
20%	100.0	21.7	100.0	46.7
28%	100.0	24.3	100.0	78.5
38%	100.0	24.3	100.0	78.5
50%	100.0	77.5	100.0	91.0
60%	100.0	83.5	100.0	100.0
70%	100.0	88.1	100.0	100.0
80%	100.0	100.0	100.0	100.0
Scarcity of providers				
20%	0.39	0.94	0.43	0.83
28%	0.42	0.92	0.47	0.65
38%	0.42	0.92	0.47	0.65
50%	0.66	0.77	0.55	0.55
60%	0.70	0.73	0.58	0.50
70%	0.71	0.71	0.61	0.49
80%	0.73	0.65	0.60	0.47
Travel distance, miles				
20%	4.45	38.93	0.44	19.15
28%	4.55	37.93	0.44	12.61
38%	4.55	37.93	0.44	12.61
50%	4.55	24.10	0.45	8.81
60%	4.55	23.28	0.45	6.97
70%	4.55	21.52	0.45	5.06
80%	4.56	20.18	0.45	3.80

Access to preventive dental care among privately insured children was negligibly affected by increases in dentists’ participation in public insurance: overall, median met need was 100% at all levels of participation in public insurance. An increase of participation in public insurance from 20% to 80% would result in an increase of the median travel distance from 0.71 to 0.74 miles, and an increase of provider scarcity from 0.42 to 0.65.

When we held other variables constant in the optimization model, an increase from 20% to 75% in the public insurance caseload of dentists currently accepting public insurance patients also increased access to preventive dental care among publicly insured children. Met need increased from 24.0% to 98.1% overall, from 17.4% to 79.6% in rural census tracts, and from 26.2% to 100% in urban census tracts. Overall, travel distance decreased from 24.8 to 10.4 miles, and provider scarcity decreased from 0.85 to 0.70. For privately insured children, the effect of an increase in the public insurance caseload from 20% to 75% was again negligible: median met need was 100%, median travel distance increased from 0.71 to 0.77 miles; and provider scarcity increased from 0.36 to 0.54.

Access measures varied overall and for urban and rural census tracts when maximum allowed travel distance for people with a personal vehicle was varied from 30 miles to 60 miles. Overall, at the state level, the percentage of met need was 76.4% for publicly insured children and 100% for privately insured children for all levels of the maximum allowed travel distance. An increase of the parameter from 30 miles to 60 miles would result in an increase of the median travel distance from 15.1 miles to 25.51 miles for publicly insured children; an increase in this parameter would not affect the median travel distance for privately insured children (0.71 miles for every value of the parameter). Median provider scarcity was equal to 0.73 for public insured children and varied from 0.46 and 0.47 for privately insured children.

## Discussion

Approximately 60% of the 2.6 million children living in Georgia are eligible for public dental insurance. We found that these children had significantly less access to preventive dental care than privately insured children and that disparities in access were most pronounced in rural areas. Our model predicted that publicly insured children would travel at least 20 miles more to a dentist’s office than would higher-income or privately insured children in 40% of all census tracts in Georgia, in 50% of rural census tracts, and in 35% of urban census tracts.

Increasing dentists’ participation rate in public insurance from its current level of 27.9% to 50% could decrease the 1-way travel distance for a dental visit for publicly insured children from 40 to 25 miles in rural census tracts and from 12 to 10 miles in urban census tracts. The finding that almost doubling dentists’ participation in public insurance would negligibly affect the access of privately insured children suggests that dentists’ patient caseload capacity could increase if the public insurance program in Georgia were to provide incentives (eg, increased reimbursement rates) for dentists to participate. Although an analysis of national data found that increasing Medicaid dental care reimbursement rates had only a modest effect on use of dental care among Medicaid-enrolled children (19,20), another study in Connecticut found that increasing Medicaid reimbursement rates from roughly 35% of the private insurance reimbursement rate to 70% during a 4-year period increased dental care use from 42% to 76% (21). The larger effect of increased reimbursement on Medicaid use in Connecticut may have been due to the state’s simplification of Medicaid administrative procedures and the raising of reimbursement rates during a recession, which could have lowered private demand and opened dental care capacity.

However, incentivizing public insurance participation by increasing dental fees may not be feasible in the current economic environment. We found that only at a public insurance participation rate of 80%, which is challenging to attain, would all need be met for preventive dental care among publicly insured children. Another potential way to increase capacity for preventive dental care is to allow dental hygienists to provide preventive dental care in school settings (22). This approach could also lead to a decrease in costs, because the marginal rate for a hygienist is less than the rate for a dentist. Because of long travel distances in rural census tracts regardless of the participation rates of dentists in public insurance, the provision of preventive dental care in schools might be an attractive solution (23). Recently, Georgia passed legislation (HB-154) to allow dental hygienists to provide this care (24).

Our estimates are more conservative than estimates produced in a recent study (25), which found that 94% of children live within 15 minutes of a dentist that accepts Medicaid. The main reasons for this difference are that we 1) accounted for the fact that dentists who accept public insurance do not devote 100% of their capacity to public insurance–enrolled children, 2) assumed that not all dentists take new public insurance–enrolled patients, and 3) focused only on access to preventive care. Optimization models such as ours have been compared with the classic catchment area method (26); although the optimization model is more complex, it has several advantages in providing more accurate access estimates (27).

Limitations of this study pertain to assumptions made to estimate access and to the limited availability of detailed data. Limitations exist in estimating need and supply for preventive dental care (10). First, we used household income thresholds for public insurance programs to estimate the numbers of children who are eligible for public insurance. Second, we relied on Georgia Board of Dentistry data to identify practice locations of dentists. Although many dentists practice from various offices, only the business address is provided by the Georgia Board of Dentistry. Third, we used the IKN database to identify dentists accepting public insurance, assuming capacity for public insurance to be within a given range. The IKN database can be inconsistent in that it includes duplicate entries, and it names many providers not found in the Georgia Board of Dentistry data. We assumed all dentists in an office accepting public insurance took publicly insured children. We also assumed that dentists accepting public insurance took all types of public insurance. Fourth, we estimated matches between patients and dentists assuming a centralized framework; we showed elsewhere (18) how the model could be modified to incorporate decentralized decision making with patients maximizing their own welfare. Finally, travel distances do not account for potential differences in the associated travel time that may arise from population density or road shape.

The methods used in this study could help decision makers identify areas where disparities in access to preventive dental care are largest and implement strategies to increase dental care capacity for public insurance patients accordingly. Without access to preventive dental care it is likely that many of these children would develop dental caries. Dental caries is one of the most common diseases of childhood (28), and effective interventions exist to prevent it (29,30). Furthermore, evidence suggests that increasing access to effective preventive dental services could be cost saving to the Centers for Medicare & Medicaid Services (31).
